# Supplementary figures and images for: Two-dimensional shear wave elastography and ultrasound-guided attenuation parameter for progressive non-alcoholic steatohepatitis
Source: PLoS One. 2021 Apr 7;16(4):e0249493. doi: 10.1371/journal.pone.0249493 (PMC8026049; doi:10.1371/journal.pone.0249493)

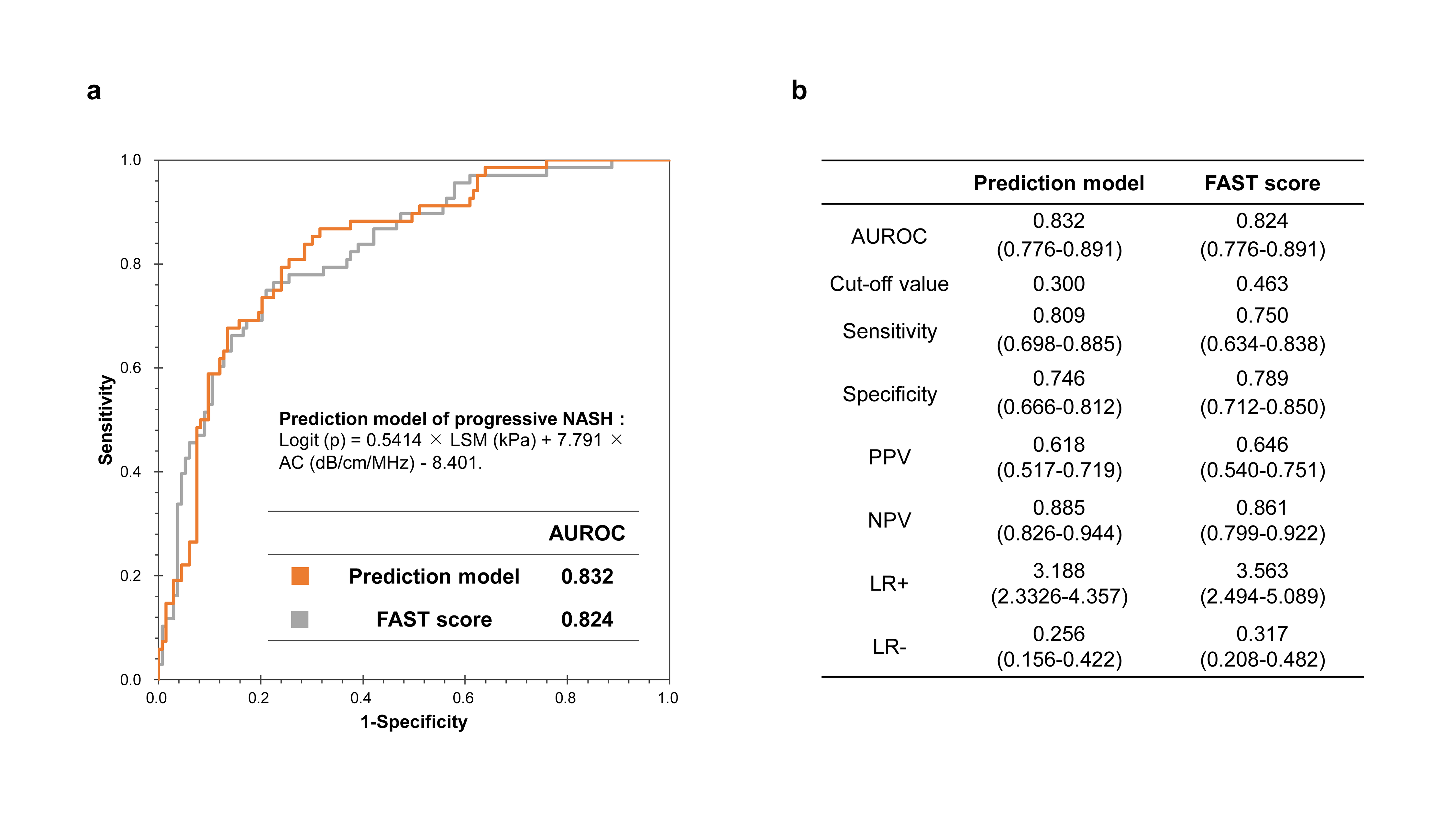

Supplement: S1 Fig — The colored box (center) represents the elastogram, and the circle (yellow) represents the region of interest where the elastic modulus (liver stiffness measurement) of the liver is acquired. The blue color indicates soft liver tissue, as semi-quantitatively presented by the color scale to the left. (TIF) [file pone.0249493.s001.tif]

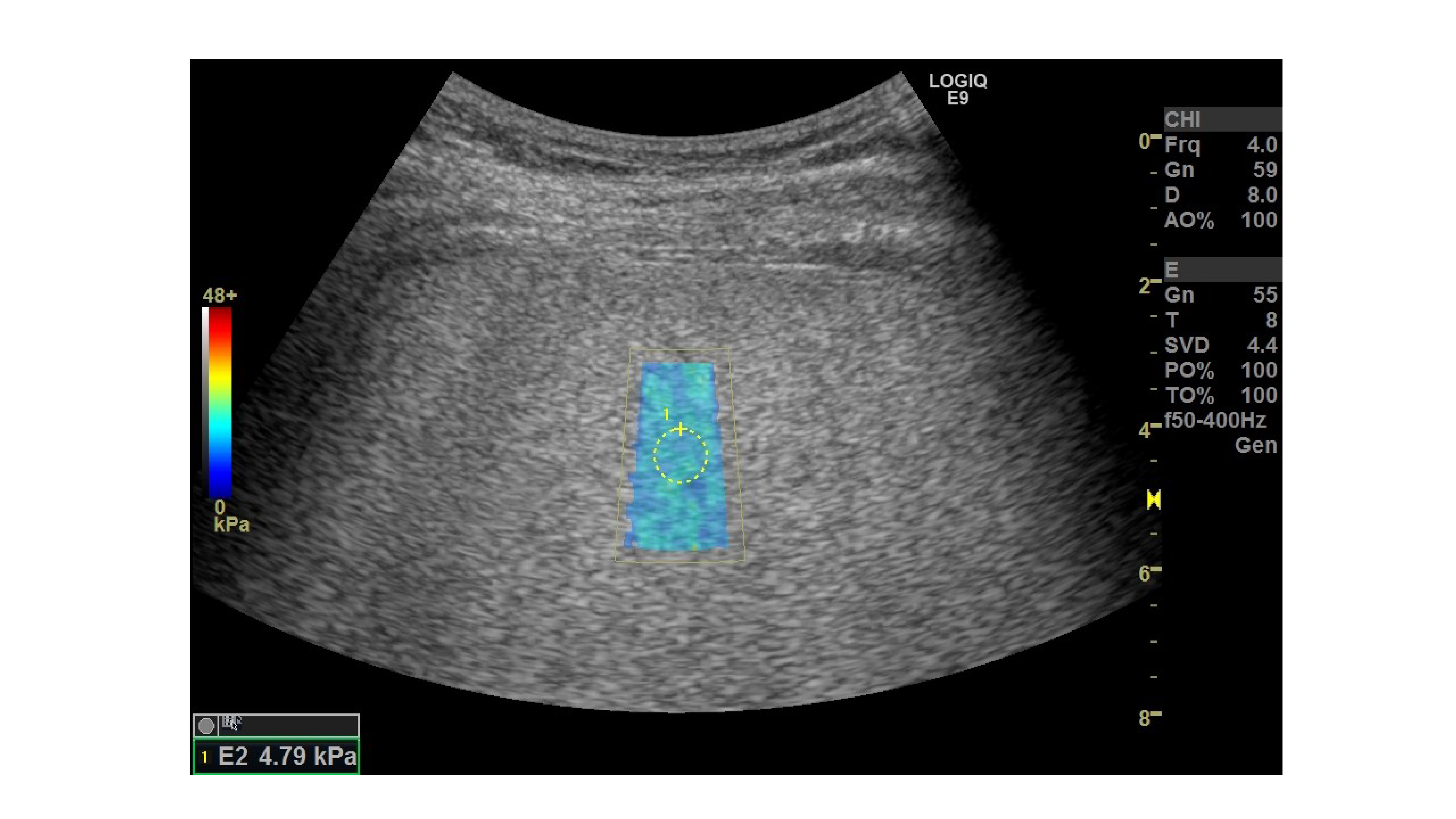

Supplement: S2 Fig — The attenuation coefficient (AC) was calculated based on the reference phantom method reported by Yao et al. [24]. This method utilizes an ultrasound phantom with known attenuation (AC: 0.44 dB/cm/MHz) and backscatter coefficients to compensate for the characteristics of transmission. The echo signal from the liver S0(f, x) (target) and the echo signal from the phantom, Sp(f, x) (reference) are described as −12flog10s0(f,x)sp(f,x)+αpx=α0x(1) where f is the frequency used, x is the length of the depth direction of the region of interest (ROI), and α0 and αp are the ACs of the tissue and phantom, respectively. The ultrasound system was calibrated using a specific acquisition set up (4.0 MHz of the fundamental B-mode) before the study, and the same acquisition setup was used for collecting the data for each patient. The results of the onetime calibration were used to calculate the AC. We acquired B-mode image data of the liver parenchyma (Segment 5), the same area subjected to two-dimensional shear wave elastography, vibration controlled transient elastography, controlled attenuation parameter, and liver biopsy (a). Radiofrequency-based ultrasound echo signals were analyzed by a dedicated prototype software program in MATLAB (MathWorks, Inc., Natick, MS, USA). One of three ultrasound engineers (T.O., S.N., and N.K.) opened each image and set the ROI on the liver parenchymal area, avoiding vessels, and at least 20 mm from the liver surface. The engineers were blinded to all patient data. The average of 10 consecutive scanning rasters was processed, followed by smoothing using a low-pass filter (b). The AC was calculated based on the signal’s decay slope A between 55 and 120 mm in depth using the least-squares method (c): 12flog10s0(f,x)sp(f,x)+αpx≈Ax+B(2) (TIF) [file pone.0249493.s002.tif]

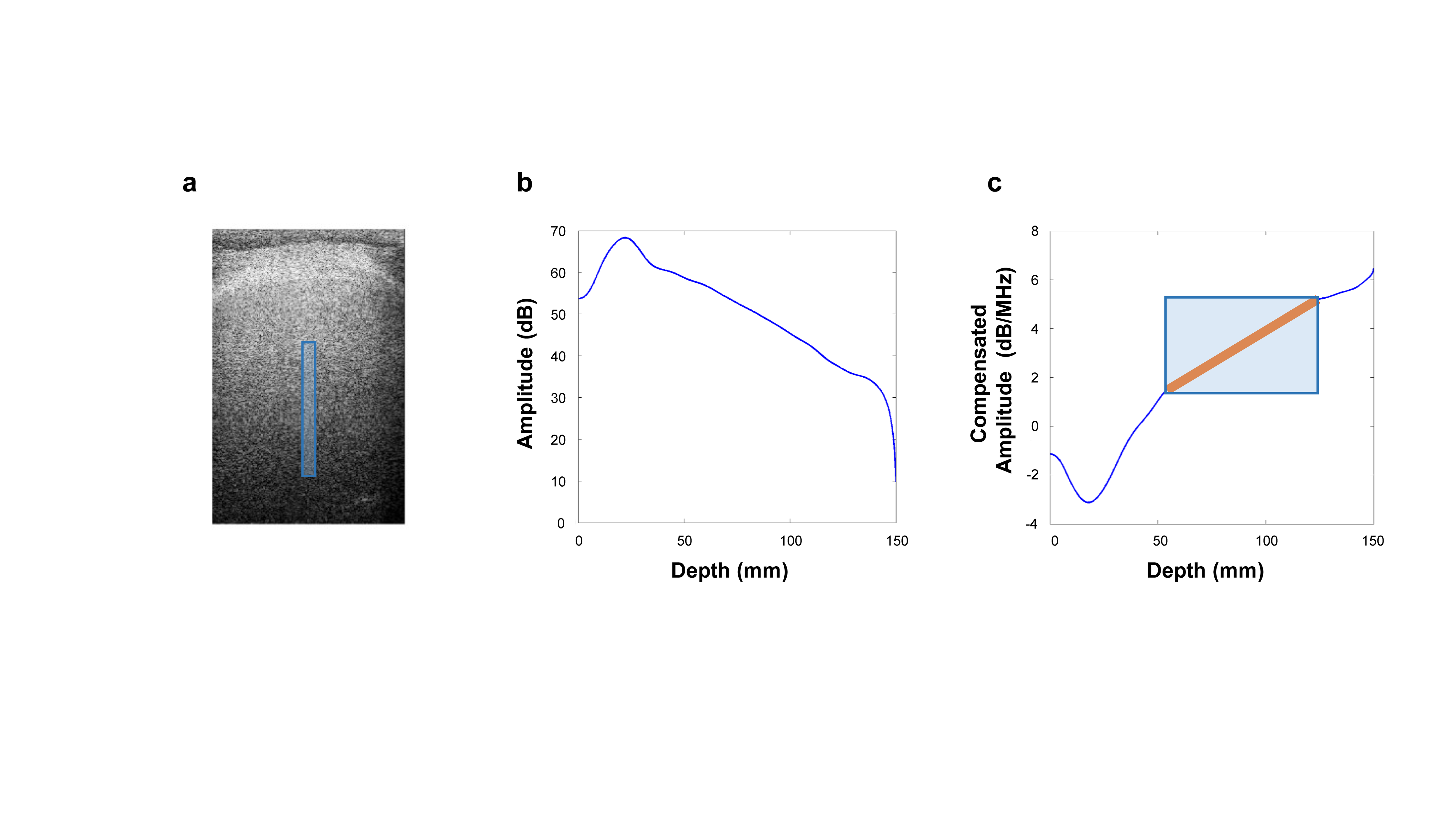

Supplement: S3 Fig — The area under the ROC curve (AUROC) for the prediction of progressive NASH was 0.832 for the prediction model, and 0.824 for the FAST score. There was no statistically significant difference between the two models. Abbreviations: AC: attenuation coefficient; AUROC, area under the receiver operating characteristic curve; FAST: FibroScan-aspartate aminotransferase; LSM, liver stiffness measurement; LR+, positive likelihood ratio; LR-, negative likelihood ratio NASH, non-alcoholic steatohepatitis; NPV, negative predictive value; PPV, positive predictive value. (TIF) [file pone.0249493.s003.tif]
