# Supplementary material for: Two-dimensional shear wave elastography and ultrasound-guided attenuation parameter for progressive non-alcoholic steatohepatitis
Source: PLoS One. 2021 Apr 7;16(4):e0249493. doi: 10.1371/journal.pone.0249493 (PMC8026049; doi:10.1371/journal.pone.0249493)
Supplement: S1 Table — (DOCX) [file pone.0249493.s004.docx]

**S1 Table. 2D SWE and VCTE measurement results by each histopathologic evaluation**

|  |  | **2D SWE** | | | **VCTE** | | |
| --- | --- | --- | --- | --- | --- | --- | --- |
|  |  | **LSM (kPa)** | | **P value** | **LSM (kPa)** | | **P value** |
| **Fibrosis stage** | |  | |  |  | |  |
|  | F0 | 6.04 | [4.89-7.75] | < 0.0001 | 7.86 | [5.93-10.53] | < 0.0001 |
|  | F1 | 6.81 | [5.22-8.46] |  | 8.16 | [6.08-11.26] |  |
|  | F2 | 8.56 | [6.99-10.46] |  | 9.56 | [7.40-14.91] |  |
|  | F3 | 10.42 | [8.91-11.33] |  | 14.90 | [12.43-17.33] |  |
|  | F4 | 14.59 | [11.18-16.43] |  | 23.66 | [17.53-26.27] |  |
| **Steatosis grade** | |  |  |  |  |  |  |
|  | S1 | 7.97 | [6.12-10.68] | 0.148 | 11.27 | [7.56-15.59] | 0.111 |
|  | S2 | 8.17 | [5.81-10.39] |  | 9.27 | [6.36-14.76] |  |
|  | S3 | 5.88 | [5.23-8.98] |  | 8.50 | [5.23-14.78] |  |
| **Lobular inflammation grade** | |  |  |  |  |  |  |
|  | A0 | 6.43 | [5.55-7.97] | 0.008 | 8.85 | [7.05-12.12] | 0.009 |
|  | A1 | 7.40 | [5.79-10.41] |  | 9.52 | [6.86-14.91] |  |
|  | A2 | 9.94 | [7.32-12.31] |  | 14.43 | [7.82-19.19] |  |
|  | A3 | 7.40 | [7.19-8.12] |  | 7.95 | [7.45-9.75] |  |
| **Ballooning grade** | |  |  |  |  |  |  |
|  | B0 | 6.03 | [4.88-6.95] | < 0.0001 | 7.87 | [6.01-10.32] | < 0.0001 |
|  | B1 | 9.61 | [7.49-11.58] |  | 13.57 | [8.11-18.44] |  |
|  | B2 | 8.47 | [6.81-10.94] |  | 9.12 | [7.88-15.13] |  |
| **Control** | | 4.61 | [4.31-6.45] |  | 5.48 | [4.11-6.45] |  |

The values are shown as the median [25-75th percentile]. 2D SWE, two-dimensional shear wave elastography; VCTE, vibration controlled transient elastography; LSM, liver stiffness measurement.

The Kruskal-Wallis test was used to compare medians among multiple comparison groups.
