# Supplementary material for: Two-dimensional shear wave elastography and ultrasound-guided attenuation parameter for progressive non-alcoholic steatohepatitis
Source: PLoS One. 2021 Apr 7;16(4):e0249493. doi: 10.1371/journal.pone.0249493 (PMC8026049; doi:10.1371/journal.pone.0249493)
Supplement: S2 Table — (DOCX) [file pone.0249493.s005.docx]

**S2 Table. UGAP and CAP measurement results by each histopathologic evaluation**

|  |  | **UGAP** | | | **CAP** | | |
| --- | --- | --- | --- | --- | --- | --- | --- |
|  |  | **AC (dB/cm/MHz)** | | **P value** | **CAP values (dB/m)** | | **P value** |
| **Fibrosis stage** | |  | |  |  | |  |
|  | F0 | 0.56 | [0.48-0.64] | 0.0001 | 240.32 | [207.19-273.86] | 0.0001 |
|  | F1 | 0.67 | [0.58-0.73] |  | 288.15 | [262.21-305.87] |  |
|  | F2 | 0.65 | [0.56-0.73] |  | 283.85 | [234.14-301.01] |  |
|  | F3 | 0.63 | [0.54-0.68] |  | 270.04 | [231.94-305.46] |  |
|  | F4 | 0.59 | [0.49-0.65] |  | 243.71 | [198.84-286.93] |  |
| **Steatosis grade** | |  |  |  |  |  |  |
|  | S1 | 0.56 | [0.49-0.63] | < 0.0001 | 243.71 | [201.09-280.44] | < 0.0001 |
|  | S2 | 0.69 | [0.66-0.73] |  | 296.31 | [283.13-325.61] |  |
|  | S3 | 0.74 | [0.72-0.79] |  | 305.34 | [287.64-321.52] |  |
| **Lobular inflammation grade** | |  |  |  |  |  |  |
|  | A0 | 0.54 | [0.48-0.61] | 0.0001 | 225.67 | [193.06-261.72] | 0.008 |
|  | A1 | 0.63 | [0.53-0.72] |  | 265.38 | [232.52-299.76] |  |
|  | A2 | 0.65 | [0.56-0.73] |  | 287.00 | [240.94-316.31] |  |
|  | A3 | 0.56 | [0.49-0.62] |  | 270.50 | [230.51-307.75] |  |
| **Ballooning grade** | |  |  |  |  |  |  |
|  | B0 | 0.55 | [0.48-0.62] | < 0.0001 | 239.02 | [197.93-272.02] | < 0.0001 |
|  | B1 | 0.60 | [0.51-0.67] |  | 271.46 | [216.52-295.86] |  |
|  | B2 | 0.70 | [0.66-0.74] |  | 305.28 | [287.43-333.81] |  |
| **Control** | | 0.44 | [0.39-0.48] |  | 196.03 | [187.32-234.32] |  |

The values are shown as the median [25-75th percentile]. UGAP, ultrasound-guided attenuation parameter; CAP, controlled attenuation parameter; AC, attenuation coefficient.

The Kruskal-Wallis test was used to compare medians among multiple comparison groups.
