# Supplementary material for: Two-dimensional shear wave elastography and ultrasound-guided attenuation parameter for progressive non-alcoholic steatohepatitis
Source: PLoS One. 2021 Apr 7;16(4):e0249493. doi: 10.1371/journal.pone.0249493 (PMC8026049; doi:10.1371/journal.pone.0249493)
Supplement: S3 Table — (DOCX) [file pone.0249493.s006.docx]

**S3 Table. Risk stratification of progressive NASH and the necessity of liver biopsy**

| **Parameter** | **Group A** | **Group B** | **Group C** | **Group D** |
| --- | --- | --- | --- | --- |
| **2D SWE**  **(Cutoff value of LSM: 6.430 kPa)** | Low | Low | High | High |
| **UGAP**  **(Cutoff value of AC: 0.493 dB/cm/MHz)** | Low | High | Low | High |
| **Percentage of NASH (%)** | 0.0%  (0/14) | 36.5%  (19/52) | 67.9%  (19/28) | 78.7%  (85/108) |
| **Percentage of progressive NASH (%)** | 0.0%  (0/14) | 7.7%  (4/52) | 35.7%  (10/28) | 50.0%  (54/108) |
| **Risk of progressive NASH** | Extremely low | Low | Medium | High |
| **The necessity of specialist referral** | Low | High | High | High |
| **The necessity of liver biopsy** | Low | High | High | Low |

2D SWE, 2D shear wave elastography; AC, attenuation coefficient; LSM, liver stiffness measurement; NAS, non-alcoholic fatty liver disease activity score; NASH, non-alcoholic steatohepatitis; UGAP, ultrasound-guided attenuation parameter.
