# Supplementary material for: Two-dimensional shear wave elastography and ultrasound-guided attenuation parameter for progressive non-alcoholic steatohepatitis
Source: PLoS One. 2021 Apr 7;16(4):e0249493. doi: 10.1371/journal.pone.0249493 (PMC8026049; doi:10.1371/journal.pone.0249493)
Supplement: Attachment — Submitted filename: Response_R1.docx [file pone.0249493.s007.docx]

March 1, 2021

Emily Chenette

Deputy Editor-in-Chief

*PLOS ONE*

Dear Editor:

We wish to resubmit our manuscript titled “Two-dimensional shear wave elastography and ultrasound-guided attenuation parameter for progressive non-alcoholic steatohepatitis” (PONE-D-21-01341) for publication in *PLOS ONE*.

We sincerely thank the editors and reviewers for their critical review, constructive comments, and valuable suggestions, which have been incorporated in our revised manuscript and enabled us to provide, what we believe, is a significantly improved account of our research. Our point-by-point responses to the comments of the reviewers are appended below. The revisions in the manuscript are denoted by underlined text, as specified.

In accordance with your instructions, we would like to update our Funding Statement as follows: This work was supported by JSPS KAKENHI [grant number JP 19K08400]. The funder provided support in the form of salaries for authors [H.K.]. However, it did not have any additional role in the study design, data collection, and analysis, decision to publish, or preparation of the manuscript. The specific roles of these authors are articulated in the ‘author contributions’ section.

In addition, we would like to update our Competing Interests Statement as follows: The authors have read the journal’s policy, and the authors of this manuscript have the following competing interests: T.O., S.N. and N.K. are paid employees of GE Healthcare Japan. This does not alter our adherence to PLOS ONE policies on sharing data and materials. The other authors declare that they have no competing interests. There are no products in development or marketing products to declare.

We thank you for your consideration and look forward to hearing from you.

Sincerely yours,

Hidekatsu Kuroda, M.D.

Division of Hepatology, Department of Internal Medicine, Iwate Medical University, Nishitokuta2-1-1, Yahaba-cho, Shiwa-gun, Iwate, 028-3694, Japan

E-mail: hikuro@iwate-med.ac.jp

Telephone: +81-19-651-5111

Fax: +81-19-907-7166

Our responses to the comments from reviewers 1 and 2 are as follows:

----------------------------------------------------------------------------------------------------------

**RESPONSES TO REVIEWER 1**

We thank the reviewer for the thoughtful and constructive review of our manuscript. We have addressed all of the reviewer's concerns in detail. Our responses to the reviewer’s comments are as follows:

**1) On page 17, it was mentioned that median LSMs obtained by 2D-SWE and VCTE in NAFLDs are correlated with liver fibrosis stages. However, the materials and methods section only mentioned that LSM was performed using 2D-SWE. How was LSM calculation using VCTE performed?**

Response:

We thank the reviewer for their comments on this point. In accordance with the reviewer's advice, we have revised the text in the Materials and methods section (Materials and methods: Pages 9–10, lines 155–172).

**2) The data of LSM scores and fibrosis stages should be listed as a table.**

Response:

We fully agree with your helpful suggestion. Accordingly, we have presented these data in a table (S1 Table, 2D SWE and VCTE measurement results by each histopathologic evaluation).

**3) The data of AC and steatosis grade as well as CAP and steatosis grade should be listed as a table.**

Response:

We fully agree with your helpful suggestion. Accordingly, we have presented these data in a table (S2 Table. UGAP and CAP measurement results by each histopathologic evaluation).

**4) Figures 2, 3a, and 3c are somehow confusing. You suggest that LSM and AC are correlated with disease progression. However, the figures indicate that LSM and AC have no correlation which may not necessarily support your findings.**

Response:

We thank the reviewer for this comment. As you pointed, we found no significant correlation between LSM using 2D SWE and AC using UGAP. However, we wish to present in Figures 2 and 3 the distribution of ultrasonic parameters according to the pathological findings of NAFLD / NASH and the usefulness of classification using two parameters. Initially, to clarify the influence of LSM and AC's interaction on progressive NASH risk, we hypothesized that combined evaluation by 2D SWE and UGAP would be useful in determining the risk of progressive NASH. We divided the diverse population of NAFLD patients into four groups, according to 2D SWE and UGAP results, and determined the difference in the distribution of NASH or progressive NASH among the four groups. Furthermore, in the logistic regression analysis, LSM and AC were independently associated with progressive NASH. We have revised the relevant text in the manuscript (Discussion: Page 30, lines 369–377).

**5) In tables 2 and 3, the alignment of each row should be adjusted. It is confusing based on the current format.**

Response:

We fully agree with the reviewer and have formatted the tables accordingly (Table 2 and 3).

**6) Did you measure 2D-SWE and UGAP in the control group? Did you incorporate the data with your NAFLD group?**

Response:

We thank the reviewer for this insightful comment. We performed 2D-SWE and UGAP analyses of the control group and described the results in the text and tables (Materials and methods: Page 7, lines 119–121; S1 Table; S2 Table.). The data of the control group were not incorporated into those of the NAFLD group.

---------------------------------------------------------------------------------------------------------

**RESPONSES TO REVIEWER 2**

We thank the reviewer for their thoughtful and constructive examination of our manuscript. We have addressed all of the reviewer's concerns in detail. Our responses to the reviewer’s comments are as follows:

**1) It’s necessary to briefly introduce the technique and examination procedure of UGAP in the Methods section.**

Response:

We thank the reviewer for raising this point. According to the reviewer's advice, we have revised the text in the Materials and methods section (Materials and methods: Pages 8–9, lines 140–153).

**2) It would be interesting to evaluate the relationship between Model for End-Stage Liver Disease (MELD) score with the two-dimensional shear wave elastography and ultrasound-guided attenuation parameter.**

Response:

We completely agree with the reviewer’s valuable suggestion. We analyzed the correlation between our proposed prediction model for progressive NASH and MELD score, finding a positive correlation (Spearman's correlation coefficient r = 0.334, P < 0.0001). We have revised the relevant text in the manuscript (Discussion: Page 32, lines 401–410).
